# Cognitive Function in Adult Offspring of Women with Gestational Diabetes–The Role of Glucose and Other Factors

**DOI:** 10.1371/journal.pone.0067107

**Published:** 2013-06-28

**Authors:** Tine D. Clausen, Erik L. Mortensen, Lone Schmidt, Elisabeth R. Mathiesen, Torben Hansen, Dorte M. Jensen, Peter Damm

**Affiliations:** 1 Department of Gynecology and Obstetrics, Hilleroed Hospital, University of Copenhagen, Copenhagen, Denmark; 2 Center for Pregnant Women with Diabetes, Department of Obstetrics, Rigshospitalet, Faculty of Health Sciences, University of Copenhagen, Copenhagen, Denmark; 3 Center for Pregnant Women with Diabetes, Department of Endocrinology, Rigshospitalet, Faculty of Health Sciences, University of Copenhagen, Copenhagen, Denmark; 4 Section of Environmental Health, Department of Public Health and Center for Healthy Aging, University of Copenhagen, Copenhagen, Denmark; 5 Section of Social Medicine, Department of Public Health, University of Copenhagen, Copenhagen, Denmark; 6 Novo Nordisk Foundation Center for Basic Metabolic Research, Faculty of Health Sciences, University of Copenhagen, Copenhagen, Denmark; 7 Faculty of Health Science, University of Southern Denmark, Odense C, Denmark; 8 Department of Endocrinology, Faculty of Health Science, University of Southern Denmark, Odense C, Denmark; University of Arkansas for Medical Sciences, United States of America

## Abstract

**Objective:**

We aimed to evaluate cognitive function in adult offspring of women with diet-treated gestational diabetes and to study potential associations with maternal glucose values.

**Materials and Methods:**

In 2003–2005 cognitive function was assessed in a cohort of 18–27 year old offspring of women with diet-treated gestational diabetes mellitus (n = 153) and offspring from the background population (n = 118). The main outcome measure was global cognitive score derived from Raven’s Progressive Matrices and three verbal subtests from the Weschler Adult Intelligence Scale. Maternal fasting- and 2-hour blood glucose values from the diagnostic oral glucose tolerance test were used as exposure variables.

**Results:**

Offspring of women with gestational diabetes mellitus had a lower global cognitive score, than offspring from the background population (93.1 vs. 100.0, P<0.001). However, when adjusted for maternal age at delivery, parity, smoking during pregnancy, pre-pregnancy overweight, family social class, parental educational level, gender, birth weight, gestational age, perinatal complications and offspring age at follow-up, the difference was no longer statistically significant. Offspring global cognitive score decreased significantly with increasing maternal fasting glucose (β = −4.5, 95% CI −8.0 to −0.9, P = 0.01) and 2-hour glucose (β = −1.5, −2.9 to −0.2, P = 0.03) in univariate general linear models, but not when adjusted for family social class and parental educational level.

**Conclusions:**

Lower cognitive test scores in adult offspring of women with diet-treated gestational diabetes were explained by well known predictors of cognitive function, but not by maternal hyperglycaemia during pregnancy. We find it reassuring that mild intrauterine hyperglycaemia does not seem to have adverse effect on offspring cognitive function.

## Introduction

Hyperglycaemia during the fetal state has long-term effects on offspring metabolism leading to increased risk of overweight and cardiovascular disease. This has been documented in animal intervention studies [1], [2] as well as follow-up studies in human populations of varying ethnic background [3]–[7], and the pathogenesis may involve epigenetic changes of gene expression [8]. To what extent a hyperglycaemic intrauterine milieu also affects offspring cognitive function and whether a dose-response relationship can be demonstrated is however less well documented. Especially, adverse effects of gestational diabetes (GDM) on offspring future health would have enormous impact from a global perspective, as the prevalence of GDM is rapidly increasing worldwide.

Animal studies, using models of both type 1- and type 2 diabetes, show deleterious dose-dependent effects of hyperglycaemia in the mature brain resulting in reversible pathoanatomical changes in the hippocampal regions of the brain as well as impaired cognitive function in the adult diabetic animals [9]. Unfortunately, there are no animal studies which investigate the potential adverse impact of intrauterine hyperglycaemia on offspring cognitive function.

Knowledge from human studies points in very different directions, as both impaired, unaffected and improved cognitive function has been found in diabetes exposed offspring [4], [10]–[24]. Many cohorts are mixed and include offspring of women with both pre-gestational diabetes and GDM and only two studies have evaluated cognitive function in adult diabetes-exposed offspring [14], [17]. Studies have demonstrated negative associations [4], [14], [16], no association [15], [17], [18] or even positive associations [13] between estimates of maternal glucose metabolism during pregnancy and offspring cognitive function. Other studies have demonstrated a possible modifying effect of breastfeeding in the first week [25], or that adverse effects of a hyperglycaemic intrauterine environment may be mediated through preterm delivery before 34 weeks gestation [17] or through a low Apgar score [15].

This study evaluates the cognitive function in adult offspring of women with diet-treated gestational diabetes, and investigates predictors of offspring cognitive function - including the possible association between estimates of maternal hyperglycaemia and offspring outcome.

## Materials and Methods

**Figure 1:**
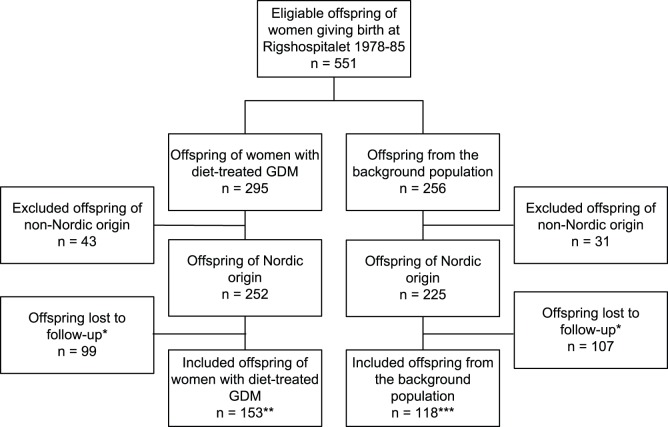
Flow chart describing subjects in the study. GDM: gestational diabetes mellitus *Reasons for lost to follow-up: Offspring of women with diet-treated GDM: 50% did not respond, 26% refused to participate, 9% had emigrated, 6% had died, 4% did not show up and 5% had other reasons. Offspring from the background population: 42% did not respond, 33% refused to participate, 9% had emigrated, 4% had died, 8% did not show up and 4% had other reasons. **Included offspring of women with GDM: 61% (153/252) of all Nordic offspring, 52% (153/295) of eligible offspring. ***Included offspring of women from the background population: 52% (118/225) of all Nordic offspring, 46% (118/256) of eligible offspring.

During 2003 to 2005 we conducted a historical follow-up study in offspring of women with diet-treated gestational diabetes mellitus (n = 295) and offspring from the background population (n = 256) (Figure 1). All subjects were 18–27 years old at follow-up and born at Rigshospitalet, Copenhagen, Denmark from 1978 to 1985. We included only the oldest singleton sibling from the study period as well as only Nordic offspring; i.e. mothers from Denmark (the great majority), Sweden, Norway or Iceland in this study of cognitive function.

From 1978 to 1985 GDM complicated 1–2% of pregnancies in Denmark [26]. The Danish routine screening procedure for GDM was, and still is, risk factor-based and not universal. In 1978 to 1985, screening was based on fasting blood glucose as well as the following risk factors: family history of diabetes, at least 20% prepregnancy overweight, previous GDM, previous delivery of a macrosomic baby (≥ 4.500 g) and glucosuria [26]. Women with risk factors and two consecutive fasting blood glucose values of at least 4.1 mmol were tested by a 3-hour 50-g oral glucose tolerance test (OGTT). GDM was diagnosed if at least two of seven glucose values exceeded the mean+3SD values for a reference group of normal weight non-pregnant women without a family history of diabetes [27]. The OGTT was defined as normal if all glucose values were below the mean+2SD values of the reference group [27]. Women with GDM were initially treated with 5000 to 8000 kJ/day diet, and additional treatment with insulin or hypoglycaemic agents was only initiated if the mean of six daily glucose determinations exceeded 7 mmol/l. Additional treatment was needed in around 20% of women with GDM. Thus, in order to study effects of mild intrauterine hyperglycaemia and to minimize the risk of including women with undiagnosed pre-gestational diabetes, we only included offspring of women with diet-treated GDM. Offspring of GDM mothers routinely had their blood glucose checked 2 hours after delivery, as well as in case of signs of hypoglycaemia and were only treated with either early feeding or intravenous glucose if necessary.

Offspring of women from the background population referred to Rigshospitalet for antenatal care and delivery were used as reference group. In the study period all women giving birth at Rigshospitalet were registered in an archive according to their own day of birth (from 1–31), and mothers in the reference group were sampled consecutively from this archive. The vast majority of these women did not have an OGTT during pregnancy due to lack of risk factors, but among those who did (n = 9) OGTT’s were within the normal range.

Coupling between mother and child was possible through the Danish Central Personal Register, and baseline data were extracted from original medical records.

Data on diabetes, overweight and the metabolic syndrome in the cohort, have previously been published [5], [6].

The protocol was approved by the local ethical committee in Copenhagen and Frederiksberg municipality (KF 11-152/04) and in accordance with the Helsinki declaration. Written as well as oral informed consent was obtained from all participants.

### Examinations at Follow-up

Offspring cognitive function was evaluated using four different well-established cognitive tests – the non-verbal Raven`s Standard Progressive Matrices and three verbal subtests (information, similarities and vocabulary) from the Danish version of Weschler Adult Intelligence Scale (WAIS) [28]–[30]. Ravens’s Progressive Matrices has been much used both as a draft board group test and as an individually administered intelligence test in clinical settings. Substantial correlations between Raven and WAIS scores [28] and between the three included subtests and the full Danish WAIS have previously been demonstrated (all r>0.79) [31]. Testers in our study were blinded with respect to exposure status, and tests were scored according to standard procedures. The intercorrelations among the four test scores were substantial (range 0.53–0.81) and consequently a composite global cognitive score was constructed. Scores from the four individual tests were linearly standardized to a mean of 100 and a standard deviation (SD) of 15 in the reference group sampled from the background population [31]. The mean of the four standardized test scores was computed and re-standardized to a mean of 100 and a SD of 15 in the background population reference group. This global cognitive score showed substantial correlations with each of the four cognitive tests included in the battery (range 0.77–0.89) and may be considered a measure of general cognitive ability.

Additionally, participants fulfilled a questionnaire regarding current parental occupation and education [32].

### Primary Outcome

Global cognitive score.

### Exposure Variables

The diabetes status (GDM vs. background population) served as a rough surrogate measure of maternal hyperglycaemia per se. Blood glucose measures (fasting and 2-hour) from the diagnostic maternal OGTT during pregnancy was used as estimates of maternal glycaemic level during pregnancy.

### Covariates/Predictors

Potential confounders and mediators were chosen based on literature and theoretical considerations.

#### Offspring

Birth weight (g), gestational age (<34, 34–36, 37–41 and ≥42 weeks), gender (male vs. female) and age at follow-up (years). Perinatal complications (yes vs. no) were defined as presence of either: placental abruption, shoulder dystocia, 5-min Apgar<7, jaundice or assisted ventilation >60 min. Neonatal hypoglycaemia (yes vs. no) was defined as treatment with intravenous glucose or at least one glucose value <2.5 mmol/l during the first two neonatal days.

#### Parents

Maternal age at delivery (years), parity (≥1 vs. 0), pre-pregnancy overweight (BMI ≥25 vs. <25 kg/m^2^) and smoking during pregnancy (yes vs. no).

We used the Danish National Institute of Social Research classification of family occupational social class [32] (similar to the British Registrar General’s Classification I-V) to indicate socio-economic position [33]. We added a social class VI representing people out of the labour market. Social class was based on information on the current occupational status of both parents, classified according to the parent with the highest position and categorized (high (I+II), middle (III+IV) and low (V+VI)). Parental educational level was based on the current educational achievement of both parents and calculated as the mean of a 1–5 index of school education and a 1–5 index of post-school training and education (1 being the lowest) [31].

We considered birth weight, gestational age, perinatal complications and neonatal hypoglycaemia as potential mediators of the association between maternal hyperglycaemia during pregnancy and offspring cognitive function.

### Statistical Analyses

Normally distributed continuous data are presented as mean (SD), whereas non-normally distributed data are presented as median (25–75 percentiles). Differences between groups were analyzed with Chi-square, Fisher’s exact, Student’s t, Mann-Whitney test or ANOVA where appropriate. We fitted general linear models using global cognitive score as outcome measure; presenting the regression coefficient (β) with 95% confidence intervals (CI), where β expresses the mean group difference measured in test-score units. In addition to univariate analyses we evaluated effects of potential confounders and mediators in seven different multivariate models. Independent predictors of offspring global cognitive score were evaluated in a model including all potential confounders and mediators (full model), and the model was not reduced.

Tests were two-tailed, with a significance level of 0.05. Data were processed using SPSS (version 20, SPSS, Chicago, IL).

## Results

### Basic Characteristics

We included 153 offspring of women with diet-treated GDM and 118 offspring sampled from the background population; representing 57% (271/477) of the offspring of Nordic origin (Figure 1). Table 1 presents baseline data on mothers and offspring in the two groups. GDM mothers were significantly older and more parous and overweight. The GDM offspring were born eight days earlier than offspring from the background population, but the majority was delivered at term in both groups and only two GDM offspring were born before 34 weeks gestation (Table 1).

**Table 1:** Data on offspring of women with diet-treated gestational diabetes compared with offspring from the background population.

	Offspring of women		
	With diet-treatedGDM	From the backgroundPopulation	P*
n	153	118	
**Maternal data at baseline**			
Age at delivery (years)	29.5 (5.4)	27.5 (4.3)	**0.001**
Parity (≥1 partus)	57% (87/153)	41% (48/118)	**0.008**
Pre-pregnancy BMI≥25 kg/m^2^	38% (58/153)	9% (10/117)	**<0.001**
Smoking during pregnancy	39% (59/131)	50% (52/104)	0.09
**Offspring data at baseline**			
Male	54% (83/153)	48% (56/118)	0.3
Birth weight (gram)	3405 (539)	3461 (481)	0.4
Gestational age (days)†	273 (269–277)	281 (275–287)	**<0.001**
Preterm delivery (<37 gestational weeks)	9% (13/153)	4% (5/117)	0.2
Very preterm delivery (<34 gestational weeks)	1% (2/153)	0% (0/117)	0.2
Perinatal complications‡	16% (25/153)	11% (13/118)	0.2
**Data at follow-up**			
Offspring age (years)	21.5 (1.9)	22.8 (2.2)	**<0.001**
Family social class	Overall: **<0.001**		
High	38% (58/153)	53% (63/118)	
Middle	38% (58/153)	41% (48/118)	
Low	24% (37/153)	6% (7/118)	
Parental educational level (1 to 5, 1 being lowest)	3.0 (1.2)	3.5 (1.0)	**<0.001**
Offspring cognitive function			
Global cognitive score•	93.1 (15.1)	100 (15)	**<0.001**
Vocabulary (subtest score)	93.6 (14.2)	100 (15)	**<0.001**
Information (subtest score)	94.0 (14.3)	100 (15)	**0.001**
Similarities (subtest score)	92.8 (15.2)	100 (15)	**<0.001**
Raven (subtest score)	96.2 (16.7)	100 (15)	0.056

Data are mean (SD) or proportions (n) if not otherwise stated. For some of the variables, numbers are changing due to missing data. Bold P<0.05. Gestational diabetes (GDM).

* Analyses of differences between means, medians and proportions were by Student’s t-test, Mann-Whitney, Chi^2^ or Fishers exact test, respectively.

† Data are given as median (25–75% percentiles), as data were not normally distributed.

‡ Defined as presence of either: placental abruption, shoulder dystocia, 5-min Apgar<7, jaundice or assisted ventilation >60 minutes.

•Defined as the re-standardized mean test score of the four cognitive subtests.

In women with diet-treated GDM, the diagnostic OGTT was performed at median 34 weeks gestation (25–75 percentiles: 31–37) with a mean fasting blood glucose at 5.2 mmol/l and 2-hour blood glucose at 7.8 mmol/l. Screening indications were: family history of diabetes (31%), ≥20% pre-pregnancy overweight (29%), previous GDM (9%), previous delivery of a macrosomic baby (6%) and glucosuria (40%); 21% had more than one risk factor.

Only nine (6%) offspring of women with GDM had neonatal hypoglycaemia, and their global cognitive score did not differ from offspring without neonatal hypoglycaemia (94.7 vs. 93.0, p = 0.7). This covariate was therefore not entered in the multivariate analysis.

Participants and subjects lost to follow-up were comparable regarding baseline data (Table 1), OGTT screening indications and timing, level of maternal glucose during the OGTT as well as incidence of neonatal hypoglycaemia (data not shown).

### Data at Follow-up

At follow-up GDM offspring were slightly younger and their parents had a lower social class and a lower educational level (Table 1).

Overall offspring of women with GDM had a significantly lower global cognitive score compared with offspring form the background population (93.1 vs. 100, P<0.001), and the same pattern was observed in the subtests of cognitive function (Table 1).

Accordingly, the global cognitive score was significantly lower in GDM offspring compared with the background population in the univariate linear regression analyses (β = -6.9, 95% CI −10.6 to −3.3, P<0.001), and this was also the case when adjusted for maternal age at delivery, parity, smoking during pregnancy, gender, offspring age (Model 1), birth weight, gestational age (Model 2), perinatal complications (Model 3), maternal pre-pregnancy overweight (Model 4) and family social class (Model 5). However, when adjusted for parental educational level (Model 6) or all potential confounders and mediators in Model 1 to 6 (Full model) the global cognitive score no longer differed significantly between the two groups (β−0.8, 95% CI −4.5 to 2.9, P = 0.7) (Table 2).

**Table 2:** Global cognitive score in offspring of women with diet-treated GDM (n = 153) compared with offspring from the background population (n = 118).

Model	ß (95% CI)	P value
Univariate	−6.9 (−10.6 to −3.3)	**<0.001**
1	−7.1 (−10.8 to −3.4)	**<0.001**
2	−6.4 (−10.2 to −2.7)	**0.001**
3	−6.7 (−10.4 to −3.1)	**<0.001**
4	−4.2 (−7.9 to −0.4)	**0.03**
5	−4.0 (−7.4 to −0.7)	**0.02**
6	−2.7 (−5.9 to 0.4)	0.09
Full model	−0.8 (−4.5 to 2.9)	0.7

Linear regression analyses giving data on global cognitive score in the offspring. Test-score mean difference is given as regression coefficient (ß) with 95% confidence interval (CI) and P value.

Bold P<0.05.

Model 1: Maternal age at delivery, parity, smoking during pregnancy, gender, offspring age.

Model 2: Birth weight, gestational age.

Model 3: Perinatal complications.

Model 4: Maternal pre-pregnancy BMI≥25.

Model 5: Family social class.

Model 6: Parental educational level.

Full model: All covariates from model 1–6.

When associations between maternal glucose values during the diagnostic OGTT (fasting or 2-hour glucose) and the global cognitive score in GDM offspring were evaluated, essentially the same pattern was seen (Table 3 and 4).

**Table 3:** Associations between maternal fasting blood glucose during OGTT and global cognitive score in offspring of women with gestational diabetes (n = 153).

Model	ß (95% CI)	P value
Univariate	−4.5 (−8.0 to −0.9)	**0.01**
1	−3.2 (−6.6 to 0.2)	0.06
2	−4.7 (−8.4 to −1.0)	**0.01**
3	−4.2 (−7.8 to −0.7)	**0.02**
4	−3.6 (−7.2 to −0.02)	**0.049**
5	−2.5 (−5.7 to 0.8)	0.1
6	−2.4 (−5.3 to 0.5)	0.1
Full model	−1.5 (−4.6 to 1.6)	0.3

Linear regression analyses giving data on global cognitive score in the offspring. Test-score mean difference is given as regression coefficient (ß) with 95% confidence interval (CI) and P value.

Bold P<0.05.

Model 1: Maternal age at delivery, parity, smoking during pregnancy, gender, offspring age.

Model 2: Birth weight, gestational age.

Model 3: Perinatal complications.

Model 4: Maternal pre-pregnancy BMI≥25.

Model 5: Family social class.

Model 6: Parental educational level.

Full model: All covariates from model 1–6.

**Table 4:** Associations between maternal 2-h blood glucose during OGTT and global cognitive score in offspring of women with gestational diabetes (n = 153).

Model	ß (95% CI)	P value
Univariate	−1.5 (−2.9 to −0.2)	**0.03**
1	−1.6 (−2.9 to −0.2)	**0.02**
2	−1.6 (−3.0 to −0.1)	**0.03**
3	−1.4 (−2.8 to −0.01)	**0.049**
4	−1.7 (−3.0 to −0.3)	**0.01**
5	−0.9 (−2.1 to 0.3)	0.2
6	−1.0 (−2.1 to 0.1)	0.08
Full model	−0.9 (−2.1 to 0.4)	0.2

Linear regression analyses giving data on global cognitive score in the offspring. Test-score mean difference is given as regression coefficient (ß) with 95% confidence interval (CI) and P value.

Bold P<0.05.

Model 1: Maternal age at delivery, parity, smoking during pregnancy, gender, offspring age.

Model 2: Birth weight, gestational age.

Model 3: Perinatal complications.

Model 4: Maternal pre-pregnancy BMI≥25.

Model 5: Family social class.

Model 6: Parental educational level.

Full model: All covariates from model 1–6.

### Predictors of Global Cognitive Score

In the univariate analyses strong predictors of offspring global cognitive score were: maternal overweight (β = −10.6, 95% CI −14.7 to -6.5, P<0.001), family social class (high: β = 18.9, 14.2 to 23.7, P<0.001 and middle: β = 8.0, 3.1 to 12.8, P = 0.001, using low social class as reference) and parental educational level (β = 7.9, 6.6 to 9.3, P<0.001). These three covariates were very closely correlated, and in analyses entering all three covariates, parental educational level was the only remaining statistically significant and by far the strongest predictor of offspring global cognitive score (β = 6.7, 95% CI 4.7 to 8.8, P<0.001). Of the other potential predictors only offspring age at follow-up (β = 1.1, 95% CI 0.3 to 2.0, P = 0.01), maternal age at birth (β = 0.6, 95% CI 0.2 to 0.9, P = 0.002) and multiparity (β = −5.8, 95% CI −9.4 to −2.1, P = 0.02) were significantly associated with offspring cognitive function in the univariate analyses, whereas: birth weight, gestational age, gender, smoking during pregnancy and perinatal complications were not.

When all potential predictors as well as the exposure variable (GDM vs. background population) were included in the model (Full model, Table 2) – significant positive predictors for global cognitive score were: parental educational level (β = 6.3, 95% CI 4.2 to 8.3, P<0.001), offspring age at follow-up (β = 1.1, 95% CI 0.3 to 1.8, P = 0.008), maternal age at delivery (β = 0.4, 95% CI 0.1 to 0.8, P = 0.02) and male gender (β = 3.2, 95% CI 0.1 to 6.3, P = 0.04), whereas multiparity was a negative predictor (β = −4.9, 95% CI −8.2 to −1.6, P = 0.004). Parameter estimates were essentially unchanged when the exposure variable was omitted from the model. Parental educational level alone explained 33.8% the of the variance in global cognitive score (R^2^ = 0.338), whereas model 6 with all 11 potential predictors increased this only slightly (R^2^ = 0.411), and further adding the exposure variable hardly changed anything (R^2^ = 0.413). Maternal glucose values (fasting and 2-hour from OGTT) were not predictors, as they were not significantly associated with offspring cognitive function when potential confounders and mediators were taken into account.

### Analyses for Possible Interactions

There was no interaction between either family social class or parental educational level and diabetes status or maternal glucose values during pregnancy with respect to the effect on offspring cognitive function. We therefore found no indications that effects of GDM or maternal glucose values was moderated by family social class or education (data not shown).

## Discussion

We found lower cognitive scores in offspring of women with diet-treated gestational diabetes compared with offspring from the background population, but not when adjusted for well known confounders as parental education. Accordingly, associations between maternal glucose values during the diagnostic OGTT in pregnancy and offspring cognitive function reflected differences with respect to confounders.

### Strength and Limitations

This is the first study to assess cognitive function in adult offspring of women with GDM. It includes offspring of a large well-characterized cohort of women with diet-treated gestational diabetes as well as an internal reference group sampled from the background population. We consider it a strength that we included only diet-treated women, as inclusion of also insulin-treated women would impose a risk of including women with undiagnosed pregestational diabetes, which is a cohort with a very different profile regarding phenotype as well as glucose exposure during pregnancy. However, as it narrows the spectrum of hyperglycaemia this could also be considered a limitation. It is strength, that the study comprises information on many well known confounders as well as estimates of maternal glucose values during pregnancy. It is a further strength that the measure of socioeconomic position in the study is validated and standardised [32]. Additionally, this measure is based on parental occupation and not offspring occupation, as the latter would potentially be influenced by offspring cognitive function and age. As harmful effects of hyperglycaemia may bee mediated through preterm delivery and exclusion of offspring that were born preterm therefore would tend to underestimate potential adverse effects in the GDM-group [17], we chose to include offspring born before 37 weeks gestation. Two children in the GDM group were born very preterm (before 34 weeks gestation), both newborn had signs of maternal metabolic derangement - jaundice, polycytaemia and hypoglycaemia with need for intravenous glucose.

It is a limitation to the study that most women from the background population did not have an OGTT during pregnancy due to lack of risk factors. However, less than 1% of Danish women without risk factors for GDM have an undiagnosed GDM [34], therefore we estimate that less than one percent of women in the reference group sampled from the background population may have had an undiagnosed GDM and consequently this might only have minor impact on our results. Furthermore we have no information on the glycaemic status of women with GDM after initiation of treatment since HbA1c and home glucose monitoring were not routinely collected in the study period. These measurements would probably better reflect the maternal glucose metabolism during pregnancy than fasting and 2-hour glucose values from the diagnostic OGTT. It is an additional limitation to the study, that the cognitive assessment was based on three verbal WAIS subtests and Raven’s Progressive Matrices, and that the global cognitive score is therefore not directly comparable with full-scale WAIS intelligence quotients. The full-scale WAIS comprises 11 subtests (six verbal and five performance subtests) and provides comprehensive assessment of not only global intelligence, but also provides information on several specific cognitive functions. Because administration of the full-scale WAIS is time consuming and tiring, it has become standard practice in many clinical contexts to use only a subset of the 11 tests. When the focus – as in the present study – is not on assessment of individual patients, but on comparing relatively large groups, administering only three of the six verbal subtests should provide a sufficiently reliable measure of verbal intelligence, particularly since we included the three subtests which are generally considered the most valid. Furthermore, substantial correlations between the three included subtests and the full Danish WAIS has previously been demonstrated [31], and Raven’s Progressive Matrices ranks among the most proven and validated non-verbal tests of intelligence. Consequently, there is reason to expect that our measure of global cognitive function would show substantial correlations with more comprehensive assessments of intelligence.

We find it unlikely that the study was influenced by selection bias, as participants and subjects lost to follow-up were comparable with regard to a variety of confounders. The external validity of the reference group sampled from the background population is supported by a Danish study on social class [33] as well as previously developed Danish WAIS norms for the age group [35]. The finding of well established predictors of cognitive achievement such as parental educational level and parity, further confirms the external validity of our cohort and findings.

Finally, it is possible that the study design or the cognitive outcome measure were not sensitive enough to detect true differences between the GDM and the reference group although power calculations suggest that the sample size was sufficient to detect a group difference in global cognitive ability of about 0.3 standard deviations - corresponding to a group difference of 4.5 IQ points.

### Other Studies

When looking only at studies of cognitive function in offspring of women with GDM, studies are small and few and conclusions are diverging. Furthermore, except for the present study, progenies are followed only in early childhood and until adolescence. Four studies, from the US, Sweden, Poland and United Kingdom, found normal intelligence in offspring exposed to GDM compared with un-exposed controls when adjusted for parental socio economic position and education [11],[19]–[24]. One of the studies found negative associations between some estimates of maternal glucose metabolism and different measures of offspring behaviour and cognitive function, but the pattern of associations was ambiguous and only few of the many different studied associations were statistically significant [19]–[22]. The authors concluded, that findings may reflect that all included women were in very good metabolic control, but, subtle cognitive deficits may not be revealed until later in life. A study from Israel found lower cognitive scores and negative associations with maternal metabolism in some sub-tests but not in others among 32 GDM offspring compared with controls matched on socioeconomic status and offspring age [16], and an Italian study found delayed visual evoked potentials in offspring of GDM mothers (n = 13) compared with controls matched on gestational age and maternal age at delivery. Delay of visual evoked potentials was associated with low Apgar scores but not with maternal glucose estimates during pregnancy [15]. We did not find negative associations between Apgar score and offspring cognitive function in any of the two groups, which may reflect that all offspring had Apgar scores at five minutes above seven (data not shown). A large Swedish study, based on register data, found increased risk of neurological diagnoses, hospital admissions and malformations in GDM offspring using only maternal smoking during pregnancy as a proxy for socioeconomic status [36]. Two studies indicate that the effect of GDM may be moderated by socioeconomic position and education. A Canadian study (N = 221) found delayed speech development, but only in offspring of mothers with a short education [37], and a study from the US found that the effect of GDM on offspring cognitive function was more profound in socially deprived offspring (n = 9) [38]. A possible explanation is that well educated women are able to stimulate their offspring in a way that compensates for deleterious effects of GDM, or that less educated women are more metabolically deranged and their children therefore more severely exposed. These studies did not include information on maternal glucose values or treatment during pregnancy, which could further corroborate this hypothesis. Our study did not confirm an interaction between GDM and social class or education, which may reflect the fact that Danish public services ensure more uniform treatment of all women with GDM irrespectively of socioeconomic resources.

A German study found normal speech development in offspring of women with GDM, but interaction with breastfeeding in the first week after birth [39]. Unfortunately, information on breastfeeding was not available in our study. Finally, a study from India found better cognitive function in offspring of women with GDM compared with un-exposed controls, and furthermore a positive association between maternal glucose estimates and offspring cognitive function among the controls [13]. The latter study is interesting as women with GDM in India tend to have a high socioeconomic position in contrast to European and North American countries where women with GDM tend to be relatively socioeconomically deprived. This observation taken together with the inconsistency of findings in the different studies indicates effects of residual confounding rather than true associations between levels of maternal hyperglycaemia and offspring cognitive function; at least regarding mild hyperglycaemia as seen in relation to GDM. In line with this, a recent study - using a Mendelian randomization approach - concludes, that a causal link between glucose exposure in utero and impaired cognitive function in childhood is unlikely [40].

More pronounced maternal metabolic derangement may adversely affect offspring cognitive function; but at the moment, there are no animal studies to explore this issue.

### Conclusions

We found no indications that the lower cognitive score in adult offspring of women with mild and diet-treated gestational diabetes mellitus compared with offspring from the background population was due to the slightly elevated maternal glycaemic levels. It is reassuring, that mild hyperglycaemia in the intrauterine milieu does not seem to have deleterious effects on offspring cognitive function. The findings in other studies of positive as well as negative associations between maternal glycaemia and offspring cognitive function may reflect residual confounding.
